# Detection of *Pneumocystis jirovecii* in oral wash from immunosuppressed patients as a diagnostic tool

**DOI:** 10.1371/journal.pone.0174012

**Published:** 2017-03-30

**Authors:** Cecilie Juul Hviid, Marianne Lund, Allan Sørensen, Svend Ellermann- Eriksen, Bente Jespersen, Mette Yde Dam, Jens Frederik Dahlerup, Thomas Benfield, Sanne Jespersen, Lars Jørgen Østergaard, Alex Lund Laursen, Bissau HIV cohort study group

**Affiliations:** 1 Department of Infectious Diseases, Aarhus University Hospital, Aarhus, Denmark; 2 Department of Clinical Microbiology, Aarhus University Hospital, Aarhus, Denmark; 3 The Bissau HIV Cohort Study Group, Bandim Health Project, Bissau, Guinea Bissau; 4 Department of Nephrology, Aarhus University Hospital, Aarhus, Denmark; 5 Department of Rheumatology, Aarhus University Hospital, Aarhus, Denmark; 6 Department of Hepatology and Gastroenterology, Aarhus Aarhus University Hospital, Denmark; 7 Department of Infectious Diseases, Hvidovre Hospital, Hvidovre, Denmark; University Hospital Zurich, SWITZERLAND

## Abstract

**Background:**

Diagnosis of *Pneumocystis jirovecii* (PJ) pneumonia ordinarily requires invasive procedures that could be avoided by PCR methodologies, if these could be designed with adequate cut-off values for confounding background carriage.

**Methods:**

We designed a novel quantitative real-time PCR assay to detect the mitochondrial large subunit rRNA gene of PJ in oral washes. To benchmark levels of PJ carriage versus infection, we tested asymptomatic immunosuppressed patients including Danish (n = 88) and West African HIV-infected (n = 142) patients, renal transplant recipients (n = 51), rheumatologic patients (n = 102), patients with inflammatory bowel diseases (n = 98), and healthy blood donors (controls, n = 50). The fungal burden in patients with PJ pneumonia (PCP, n = 7) was also investigated.

**Results:**

Danish HIV-infected patients (with viremia/low CD4) and recent transplant recipients were at most risk of being carriers (prevalence of 23% and 16.7% respectively), whereas PJ was rarely detected among rheumatologic patients, patients with inflammatory bowel diseases, and untreated West African HIV patients. PJ was not detected among healthy controls. The fungal burden in patients with PCP fell rapidly on treatment.

**Conclusions:**

The quantitative PCR method described could conceivably discriminate between carriage and disease, given suitable threshold values for the former, and predict treatment efficacy by measures of the fungal burden in daily oral washes.

## Introduction

The opportunistic pathogen *Pneumocystis jirovecii* (PJ) is a well-known cause of severe Pneumocystis pneumonia (PCP) among immunosuppressed patients, particularly those infected with HIV [1, 2]. PCP also occurs in non-HIV immunosuppressed patients, including those with hematologic malignancies and transplant recipients. Recently it has become a significant opportunistic infection in patients receiving immunosuppressive medication for autoimmune or inflammatory diseases [3–5].

Microscopy of broncho alveolar lavage (BAL) fluid is still the mainstay of PJ detection in conjunction with histologic staining or immunofluorescence [6, 7], however PCR methods are also often used. The exquisite sensitivity associated with PCR may, paradoxically, be its greatest drawback. Patients may be carriers of PJ [8,9] with a prevalence of colonization in BAL fluids from HIV patients ranging from 15–44% [10,11]. Less is known about carriage among non-HIV immunosuppressed patients, although several studies (again of BAL fluids) suggest rates of 14–24% [12, 13].

PJ can also be detected in other specimens obtained by non invasive methods such as nasopharyngeal aspirates, induced sputum and oral wash. PCR methods using oral washes have been trailed in an attempt to avoid invasive procedures [14,15] given the ease with which these samples can be obtained without specialist equipment or training. Use of non-invasive specimens, however, has its caveat, since sensitivity in such specimens is lower. Thus a gradual decrease in PJ copy number has been demonstrated in induced sputum and nasopharyngeal aspirated compared with BAL [8,16].

The aims of this study were to 1) develop a quantitative PCR assay to be used with oral wash with which to estimate the frequency of carriage in different groups of immunocompromised patients and healthy bloddonors and 2) compare the fungal burden in immunosuppressed patients with and without PCP to establish a cut-off value.

## Material and methods

### Danish HIV infected patients

HIV infected patients (n = 88) from the outpatient clinic at the department of infectious diseases, Aarhus University Hospital, were included. They were divided into four groups according to viral load (below or above 1000 copies/mL) and CD4 cell count (below or above 200 cells/μL) at inclusion.

### HIV infected patients from Guinea Bissau, West Africa

Newly diagnosed HIV patients (n = 142) from the Hospital National Simão Mendes in Guinea Bissau in West Africa were included at their first consultation. Patients were divided into two groups according to CD4 count (below or above 200 cells/μL).

### Renal transplant recipients

Renal transplant recipients (n = 51) from the outpatient clinic at the department of nephrology, Aarhus University Hospital, were recruited. Twenty-five patients were examined two to six months after transplantation, with 26 patients examined more than a year after transplantation.

### Rheumatologic patients

Rheumatologic patients (n = 102) from the outpatient clinic at the department of rheumatology, Aarhus University Hospital, were included. These patients were divided into four groups defined by the immunosuppressive medication they received; the underlying diagnosis was not considered. The relevant medications were TNF-α inhibitors (n = 27), IL6 inhibitors (n = 25), rituximab (n = 25), and methotrexate (n = 25).

### Patients with inflammatory bowel disease (IBD)

Patients with IBD (n = 98) were included from the outpatient clinic at the department of hepatology and gastroenterology, Aarhus University Hospital. These patients were divided into groups based on the immunosuppressive medication received (i.e. TNF-α inhibitors, n = 50; 5-ASA preparation (5-aminosalicylic acid), n = 48).

### Healthy blood donors

Healthy blood donors (n = 50) from the blood bank, Aarhus University Hospital, were included as non-immunosuppressed controls.

### Patients with PCP

Seven patients with verified or suspected PCP were included. Four attended the department of infectious diseases, Hvidovre Hospital, Copenhagen, with the remainder at the department of infectious diseases, Aarhus University Hospital. In addition to the oral wash, BAL fluids were available from three of the seven cases.

### Quantitative PCR assay

DNA was extracted from 0.5 mL of oral wash using the MagNA Pure 96 instrument (Roche Diagnostics, Basel) and Viral Nucleic Acid Large Volume Kit according to the manufacturer’s instruction. Two TaqMan real-time PCR assays targeting PJ genes were used. One assay amplified a 121 bp fragment of the multicopy mitochondrial large subunit rRNA gene (mtSUrRNA) using the primers PjFI and PJRI (Eurofins Genomics GmbH, Ebersberg, Germany) and the probe PjPI (Applied Biosystems by ThermoFisher Scientific Waltham, MA, USA; Table 1), as described previously [8].

**Table 1:** Primers and probes.

Gene target	Sequence (5’-3’)
PjFI	CTGTTTCCCTTTCGACTATCTACCTT
PjRI	CACTGAATATCTCGAGGGAGTATGAA
PjPI	FAM-TCGCACATAGTCTGATTAT-MGB
PhHV F	GGGCGAATCACAGATTGAATC
PhHV R	GCGGTTCCAAACGTACCAA
PhHV P	CY5-TTTTTATGTGTCCGCCACCATCTGGATC-BBQ

The second assay amplified 104 bp of the singlecopy kex-1 gene from PJ using the primers kexF and kexR (Eurofins Genomics GmbH, Ebersberg, Germany), as previously described [16], with a minor primer modification (kexF: 5'-CAATCCTGTTCCAATGCCTAA-3).

The final reaction volume was 15 μL (5 μL extracted DNA + 10 μL mixture). The reaction mixture comprised the probe (0.2 μM), primers (0.2 μM of each), ddH_2_0, and 7.5 μL of the TaqMan Fast Advanced Master Mix (Applied Biosystems by ThermoFisher Scientific Waltham, MA, USA). The TaqMan real-time PCR assay was performed on a LightCycler 480II (Roche Diagnostics A/S, Hvidovre, Denmark). Amplification cycles included 10 min at 95°C followed by 45 cycles of 1 sec at 95°C, then 20 sec at 60°C. Triplicates of standards in tenfold serial dilutions from 10^−7^ to 10^−14^ were included.

As a positive control, a synthesized single stranded oligo of the 121 base pair DNA fragment generated by the PjFI and PjRI primer set (Table 1) was included as a standard. Based on the primers used, DNA sequence was identified using the BLAST database (Basic Local Alignment Search Tool), then synthesized by the American company Integrated DNA Technologies, Coralville, Iowa, USA. Dilutions of the standards were prepared in ddH_2_0 and stored at -80°C.

A negative control, consisting of ddH_2_0, was included to exclude contamination as well as a positive control (standard diluted to 10^−8^ corresponding to 3 x 10^6^ copies) to assess the sensitivity and quality of each PCR run. An internal control was added to all samples to test for the presence of PCR inhibitor. This consisted of primers and a probe for the Phocine herpes virus (PhHV) [17,18]. Inhibition was considered a possibility if the crossing point PCR cycle (cp) for PhHV PCR was over 30. The quality of the oral wash was evaluated using the housekeeper gene RNase P [19]. The TaqMan Copy Number Reference Assay, human, RNase P (Applied Biosystems by Thermofisher Scientific Waltham, MA, USA) was used according to the manufacturer’s instructions, with the exception that the reaction volume was lowered to 15 μL.

### Ethics

The study was reviewed and approved by the Ethical Committee of Region Midt.

A written consent was obtainedfrom study participants and stored together with documents related to the study. This procedure was approved by the ethical commitee.

### Data analysis and statistics

Fluorescence data were analyzed using the LightCycler software (Roche Diagnostics A/S, Hvidovre, Denmark). Cp values were obtained using the second derivative analysis mode of the LightCycler software. The automated method identifies the crossing point of a sample i.e. where the fluorescence curve of the sample turns sharply upward. This turning point will be a maximum on the second derivative plot of the reaction. The standards were used to generate a standard curve for the quantification of positive patient samples. Statistics and curves were generated with GraphPad Prism version 6.0 (GraphPad Software, San Diego, CA, USA).

## Results

### Quantification of P. jirovecii mtLSU gene by real time PCR: Sensitivity and specificity

The singlecopy gene kex1 and the multicopy gene mtLSU were selected as candidate target genes, then compared. With Cp values below 35, the PCR efficiency for the mtLSUrna and kex-1 assays were 100,1 and 98,0 respectively. Although these efficiencies were similar, mtLSU was superior with a difference of four Cp values for the same target concentration (i.e. the detection limit for mtLSU would be 16 times lower than for kex-1). Therefore mtLSU was chosen as the target gene. The relation between Cq and gene copies was linear over a range of 7,5 log (S1 Fig)

Cv values of 4 replicates of each dilution of the standard row ranged from 0,2 to 1,3 (S1 Table). Inter-assay variation of patient samples were measured using DNA from eight samples, which were run 5 times with more than a year between first and last run. Cv values ranged from 6–10% for PCR reactions containing above approximately 700 copies, and ranged from 50% to 60% for PCR reactions containing copy numbers between 20 and 30. Median and interquartile ranges are also shown in the table (S2 Table).

All negative (water) and positive controls (the standard in dilution 10^−8^) performed as expected. Inhibition of the PCR reaction occurred with only one patient, suspected to have PCP. The RNase P assay showed only minor variations between samples and standardization was not necessary. Two samples from a blood donor and a renal transplant patient were negative and were excluded.

#### Sensitivity

**Fig 1:**
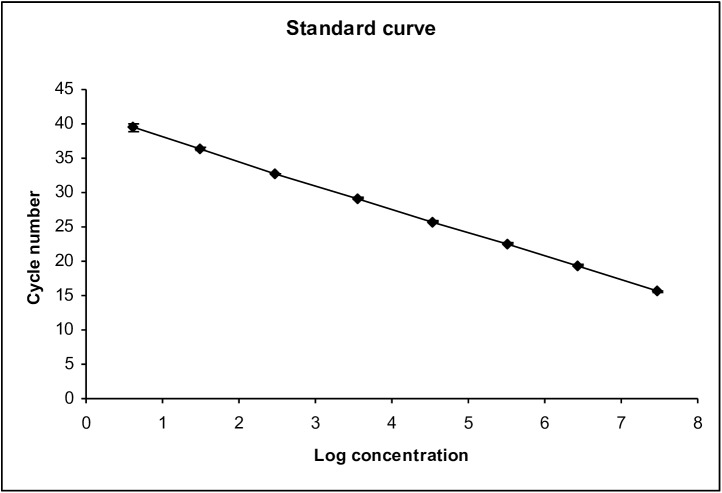
The standard curve shows linearity over a large dynamic detection range (from 3 to 30.000.000 copies) of the PCR assay. Mean and errorbars of one standard deviation are shown All negative (water) and positive controls (the standard in dilution 10^−8^) performed as expected. Inhibition of the PCR reaction occurred with only one patient, suspected to have PCP. The RNase P assay showed only minor variations between samples and standardization was not necessary. Two samples from a blood donor and a renal transplant patient were RNase P negative and were excluded.

The method was extremely sensitive, with a large dynamic detection range. The lower level of detection was 3 copies per PCR reaction with linearity in the range 3–30.000.000 copies per PCR reaction (Fig 1).

#### Specificity

The assay was tested on 36, mainly respiratory, microorganisms. The 16S and 18S-28S-rDNA intergenic spacer genes were used to confirm the presence of bacteria or fungi in these samples.

### Detection of PJ in oral fluids obtained from asymptomatic immunosuppressed patients and blood donors

#### HIV patients

HIV patients were subdivided by CD4 count (above or below 200 cells/μL) and, in Danish HIV patients, by HIV-RNA level (above or below 1000 copies/mL). All patients from Guinea Bissau were untreated, thus viral load would be above 1000 copies/mL in the vast majority of patients. Demographics are shown in detail in Table 2 with respiratory data and PCR results shown in Table 3.

**Table 2:** HIV patients. Demographics.

		Gender		Age (median—years)	Smoker (%)
		Male (%)	Female (%)		
**Danish**Low HIV RNA (<1000 copies/mL) n = 59	CD4 <200 cells/μLn = 11	7 (63.6)	4 (36.4)	47	1 (9)
	CD4 >200 cells/μLn = 48	38 (79.2)	10 (20.8)	53	13 (27.1)
**Danish**High HIV RNA (>1000 copies/mL)n = 29	CD4 <200 cells/μLn = 13	8 (61.5)	5 (38.5)	33	5 (38.5)Unknown: 3 (23)
	CD4 >200 cells/μLn = 16	13 (81.3)	3 (18.7)	42	5 (31.3)Unknown: 1 (6.3)
**African**n = 142	CD4 <200 cells/μLn = 80	53 (66.2)	27 (33.8)	38	3 (3.8)
	CD4 >200 cells/μLn = 62	35 (56.5)	27 (43.5)	36	3 (4.8)

**Table 3:** HIV patients. P. *jirovecii* data.

		Resp. sympt. (%)	Previous PCP(%)	PCP prophylaxis (%)	PCR positive(%)	*P*. *jirovecii* gene copy number/PCR reaction
**Danish**Low HIV RNA (<1000 copies/mL)n = 59	CD4 <200 cells/μLn = 11	6 (54.5)	5 (45.5)	8 (72.7)	0	
	CD4 >200 cells/μLn = 48	16 (33.3)	4 (8.3)	2 (4.2)	1 (2.1)	3 copies
**Danish**High HIV RNA (>1000 copies/mL)n = 29	CD4 <200 cells/μLn = 13	4 (30.7)	1 (7.7)	3 (23.1)	3 (23.1)	1089 copies5 copies3 copies
	CD4 >200 cells/μLn = 16	2 (12.5)	1 (6.3)	0	0	
**African**n = 142	CD4 <200 cells/μLn = 80	31 (38.8)	Unknown	0	3 (3.8)	524 copies15 copies3 copies

The HIV patients at greatest risk of being carriers had uncontrolled viremia and a low CD4 count (<200). Three of 13 Danish HIV patients with uncontrolled viremia and a low CD4 count were carriers (23%), and three received PCP prophylaxis with sulfamethoxazole-trimetoprim (SMZ-TMP), which most likely had an effect on detection of PJ. Thus, three of ten patients considered at risk for PCP, but without clinical PCP, were carriers (33%). The PCR positive patients comprised a newly diagnosed 36-year-old male with a fungal burden of 1.089 copies. This patient had a viral load of 1.061.834 copies/mL and a CD4 count of 50 cells/μL. Prior to inclusion he presented with mild respiratory symptoms but no history of PCP. He received antiretroviral therapy immediately after admission, and did not progress to clinical PCP. Only one of the patients with a low viral load and a high CD4 count was colonized (3 copies detected). Seventy-percent of non-viremic patients with a CD4 count <200 received SMZ-TMP at the time of inclusion. None of these patients were carriers.

Carriage was less frequent among the Africans at risk for PCP. Thus PJ was detected in only three percent of patients considered at risk. Only one of the PCR positive patients had a CD4 count above 200 (3 gene copies). PJ copy numbers between 3 and 524 were detected. All PCR positive patients were non-smokers and infected with HIV type 1.

#### Renal transplant recipients

Demographics are shown in detail in Table 4, with respiratory data and PCR results shown in Table 5. Patients received standard immunosuppression with basiliximab and methylprednisolone induction, followed by tacrolimus, mycophenolate mofetil, and low dose prednisolone.

**Table 4:** Renal transplant recipients. Demographics.

Time after transplantation	Gender		Age(median—years)	Smoker(%)
	Male (%)	Female (%)		
**2–6 months**n = 24	20 (83.3)	4 (16.7)	54	4 (16.7)
**> 1 year** n = 26	14 (53.8)	12 (46.2)	55	3 (11.5)

**Table 5:** Renal transplant recipients. P. *jirovecii* data.

Time after transplantation	Resp. sympt.(%)	Previous PCP(%)	PCP prophylaxis(%)	PCR positive (%)	*P*. *jirovecii* gene copy number/PCR reaction
**2–6 months**n = 24	9 (37.5)	0	3 (12.5)	4 (16.7)	59300 copies12 copies5 copies3 copies
**> 1 year** n = 26	13 (50)	0	0	0	

Carriage was seen only in patients who were transplanted less than 6 months before an oral wash was obtained, with none seen in patients on PCP prophylaxis (3). Thus four of 24 patients were colonized (16.7%). Three of these 24 patients received PCP prophylaxis. When omitting those patients, 19.5% of patients at risk were carriers. In the oral wash from one PCR positive patient, 59.300 copies were detected. At the time of his inclusion, he had experienced dyspnoea for a couple of weeks. On follow-up, his dyspnea was still present 3 weeks later, at which time immunosuppression was reduced, resulting in the disappearance of his pulmonary symptoms. He did not undergo further diagnostic procedures.

#### Rheumatologic patients

None of the patients treated with rituximab or TNF-α inhibitors were and only one (4%) of the patients treated with IL6 inhibitors was carrier (9 copies). He had respiratory symptoms and had also been prescribed 10–12.5 mg prednisolone for three months. Three (12%) of the patients with rheumatoid arthritis treated with methotrexate as monotherapy were carriers and presented with 6, 28 and 14 PJ copies.

#### Patients with IBD

None of the patients receiving TNF-α inhibitors were carriers, but one patient (2.1%) receiving a 5-ASA preparation was (3 copies). This was a 22-year-old man with no pulmonary complaints, receiving a daily dose of 25 mg prednisolone as a supplementary treatment.

#### Healthy blood donors

PJ was not detected in any of the healthy blood donors.

#### Conclusion and follow-up

Carriage was most frequently seen in renal transplant patients within the first 6 months after operation and in Danish HIV infected patients with viremia combined with a low CD4 count.

A high fungal load above 1000 copies/ml was suggestive of PCP, since two patients with respiratory symptoms improved without specific treatment after initiating antiretroviral therapy or reduction of immunosuppression. Low fungal load, however, did not suggest PCP, and no patients with a PJ copy number below 30 copies/ml developed symptomatic disease on follow up. One African HIV patient with an intermediate fungal load of 524 copies/ml was asymptomatic. Unfortunately follow up data were not available. Thus, copy numbers in in intermediate range, 30–1000 copies/ml may represent a gray zone within which PJ in oral wash cannot be used to predict incipient PCP with the present data.

### PCP sub study

Seven HIV patients with suspected or verified PCP were included. The three patients shown in Table 6 were all followed up with oral washes after initiation of treatment. It was thought highly likely that patient 1 had PCP, but due to bilateral pneumothorax, a BAL could not be performed. The patient had an untreated HIV infection with a viral load >100.000 copies/mL and a CD4 count of 10 cells/μL. Oral washes were produced on day 0, day 1, and day 4. Interestingly, a striking and rapid decrease in copy number was identified after the initiation of treatment, declining from 30.000 copies (on admission), to 925 copies (day-1), to 0 (day-4). Patient2 had an untreated HIV infection with a viral load >1.000.000 copies/mL and a CD4 count of 20 cells/μL. At the time of admission, he had dyspnoea and a cough. PJ was detected by microscopy (methenamine silver) in BAL fluid. Oral washes were produced before PCP treatment (day 0) and on days 1, 3, 4, and 5. His fungal burden decreased from 9050 copies to 0 copies after 5 days of treatment. Patient 3 also had an untreated HIV infection at the time of admission, with a viral load of >400.000 copies/mL and a CD4 count of 50 cells/μL. A PCP diagnosis was made based on the detection of PJ in BAL fluid (1920 copies), and mild clinical symptoms suggestive of early PCP. The patient had only 9 copies in oral fluid at diagnosis, declining to zero by day 3 of PCP treatment.

**Table 6:** HIV patients with PCP with follow-up.

	Gender	Age(Years)	Viral load(copies/mL)	CD4 count(cells/μL)	*P*. *jirovecii* gene copy number/PCR reaction(oral wash)	*P*. *jirovecii* gene copy number/PCR reaction(BAL fluid)
**1**	Male	29	101416	10	Before treatment: 300.000 copiesDay 1 on treatment: 925 copiesDay 5 on treatment: 0 copies	Not done
**2**	Male	66	1.279477	20	Before treatment: 9050 copiesDay 1 on treatment: 190 copiesDay 3 on treatment: 38 copiesDay 4 on treatment: 7 copiesDay 5 on treatment: 0 copies	Not done
**3**	Female	44	433506	50	Before treatment: 9 copiesDay 1 on treatment: 7 copiesDay 3 on treatment: 0 copies	1920 copies(Prior to treatment)

Data from the four HIV patients that were not followed up with oral washes after initiation of treatment are shown in Table 7. All had viremia and a low CD4 count on admission. Unfortunately, oral washes were obtained after the initiation of treatment, hence the number of PJ copies in oral fluids were low, in accordance with our previous findings.

**Table 7:** HIV patients with PCP without follow-up.

	Gender	Age (Years)	Viral load (copies/mL)	CD4 count (cells/μL)	*P*. *jirovecii* gene copy number/PCR reaction (oral wash)	*P*. *jirovecii* gene copy number/PCR reaction (BAL fluid)
**1**	Male	33	15001	6	3 copies (Two days treatment)	2278 copies (Two days treatment)
**2**	Male	46	15001	0	3 copies (Four days treatment)	266500 copies (Four days treatment)
**3**	Male	34	2.980000	81	374 copies (Two days treatment)	Not done
**4**	Female	47	649000	2	3 copies (Four days treatment)	Not done

## Discussion

### Quantitative PCR assay

The quantitative PCR assay amplifying the mtLSUrRNA gene from oral washes, had a high specificity and sensitivity, with a detection range from 3 to 30.000.000 copies. Standardization of the oral samples was not needed given the homogeneity of the oral wash. Nor was inhibition of the PCR reaction a problem using these samples.

In the selection of a target gene a single copy gene, kex1, was compared with a multi copy gene, mtLSUrRNA [8, 14,20,21], with the latter found to be superior, most likely due to a higher number of gene copies. A quantitative PCR method based on the mtLSUrRNA gene has also been developed by others and found to be superior compared to other multicopy genes, such as the major surface glycoprotein gene (MSG) [21–22].

### Carriage

Carriage with PJ was primarily seen in Danish HIV infected patients (with viremia and a low CD4 cell count, and renal transplant patients 2–6 months after transplantation. In contrast, PJ was detected in only three percent of HIV infected patients from Guinea Bissau, at risk of PCP. Carriage in iatrogenically immunosuppressed patients was rare and most commonly occurred when prednisolone was added to the regimen. PJ was not detected in healthy controls.

A comparison of the prevalence of PJ colonization with other studies is difficult to perform with any surety given the different populations tested, analyses of different types of respiratory specimen, and the use of different diagnostic techniques with differing sensitivities. Several studies analyzing BAL fluids and induced sputum were also unable to detect PJ in immuno-competent individuals [10, 22,23] in accordance with our results. In contrast, Medrando et al. [24] analyzed oral washes from health care workers with a qualitative nested PCR design and detected PJ in 20% of immuno-competent individuals.

Carriage in oral washes from asymptomatic HIV-infected patients has, to our knowledge, not been studied previously. Investigating BAL fluids and induced sputum from patients with a CD4 count below 400, Leigh et al. [10] detected PJ in 20% of specimens, in agreement with our study. In contrast, Morris et al. [25] detected PJ in 46.2% of material from lung autopsies, and found no association with CD4 cell count. Botterel et al examined BAL fluids from a large group of immunosuppressed patients (HIV and non-HIV) and found 17% to be positive by PCR and negative by immunofluorescence. None of these developped PCP, however a higher 1 year mortality was noted among patients with inflammatory disorders [26]. Mühlethaler et al identified a cut off level of PJDNA level above which PCP could be diagnosed with certainty and could exclude PCP with a combination of clinical information and negative PCR result. However, a relative large gray zone was identified within which carriage and disease could not be determined [27]. Other studies, investigating BAL fluids and induced sputum, recorded a prevalence of 15–44% [10, 12, 13]. Alanio et al. [8], the sole investigators to use a quantitative design, recorded a prevalence of (15%).

Few studies have dealt with carriage in HIV infected patients from sub-Saharan Africa. Jensen et al. [28] found oral wash specimens positive for PJ in only one HIV patient among 94 with CD4 counts below 200, half of whom were on PCP prophylaxis. In our study, the prevalence of PJ carriage was significantly lower in the HIV infected patients from Guinea Bissau compared to Danish patients, suggesting that PJ and PCP are less prevalent in this region. In accordance with our results, van Oosterhout et al. [29] investigated the incidence of PCP in HIV infected patients in Malawi and found the incidence to be low compared to other pulmonary infections. Other studies from Ethiopia [30] and Malawi [31] found higher incidences of PCP (29.7% and 27% respectively) suggesting regional differences.

Among the renal transplant recipients, carriage was only present in those recently transplanted (19.5%). To our knowledge, no previous studies have been performed on oral washes from this population. Fritzsche et al. [32] reported PJ colonization in 18.6% of renal transplant recipients when investigating induced sputum, and found a higher rate of carriage in those transplanted more than 2 years previously, contrary to our results, which suggested that this was only seen in the first 6 months after transplantation. Guigue et al analyzed nasopharyngeal swaps from a mixture of immunocompromised patients and found 16 patients (4,8%) PCR positive for PJ, 6 of whom with PCP as the only pathogen. Five of these patients were treated for PCP, suggesting that detection of PJ as the only pathogen in in this type of patients represents a high risk of PCP [16].

In studies on deep airway specimens from a mixed group of non-HIV immunosuppressed patients (hematological malignancies, solid organ transplantation, hemolytic anemia, solid malignancies, autoimmune diseases, sarcoidosis, and other lung diseases treated with steroids) a prevalence of 14–24% [8, 12] was found. In our study PJ carriage was infrequent in iatrogenically immunosuppressed patients other than those transplanted and being prescribed prednisolone.

Interestingly 12% of the patients with rheumatoid arthritis treated with methotrexate without concomitant steroid were colonized. A Japanese study [33] reported similar findings. Induced sputum was investigated with 10.9% of rheumatoid arthritis patients treated with low-dose methotrexate colonized. It was notable however that 50% of these patients also received 5 mg/day of steroid.

We identified a single IBD patient colonized with PJ. This patient was treated with ASA and a moderate dose of prednisolone, whereas no patients on anti-TNF α were colonized. Long et al. reported a hazard ratio of three for IBD patients, with prednisolone being a major risk factor [34].

PCP is normally diagnosed by demonstrating PJ by immunofluorescence or histological stains in material obtained from deep airways by BAL. Qualitative or quantitative PCR, however, is often used, but as mentioned above with some uncertainty, since carriage has been demonstrated in immunocompromised patients and applying a qualitative method a gray zone is often seen, within which it is not possible to differentiate disease from carriage. Furthermore, a study from Valero et al [35] suggests that copy number of PJ does not accurately reflect fungal load detected by microscopy, but may depent on the PJ gene used for amplification.

Since BAL is an invasive procedure and may be associated with a risk of respiratory deterioration, detection of PJ by a PCR method in material obtained with non-invasive methods such as oral wash is of interest to diagnose PCP. It is, however, important to emphasize that a negative result cannot necessarily rule out PCP in symptomatic patients, opposite a similar finding in BAL. On the other hand, detection of PJ in oral wash suggest active proliferation in the lungs and may support PJ as a possible aethiologic agent. The quantitative PCR test described in this study is potentially a diagnostic tool for PCP provided an appropriate cut off level is chosen. Such a level may vary between different groups of immunocompromised patients. Furthermore choice of gene for amplification will influence results, since amplification of a multicopy gene will increase sensitivity. Thus HIV infected patients are known to present with a higher fungal burden than other groups of immunocompromised patients. Our results suggest that patients with a high fungal load–here defined as more than 1000 PJDNA copies/ml may represent incipient or acute PCP, whereas patients with a low fungal burden (below 30 copies/ml) were asymptomatic and did not develop PCP on follow-up. Our findings must now be confirmed in a larger study in these groups of patients to establish a reasonable cut off value, however, it seems that in patientstreated with TNF-α inhibitors and IL-6 inhibitors, a positive PCR result might be included in a diagnostic strategy.

### PCP substudy

Oral washes from three PCP patients were obtained before treatment and consecutively on the following days. After initiation of PCP treatment, the fungal burden in the oral washes fell rapidly. Thus, ideally, specimens must initially be obtained prior to treatment. Oral washes from other PCP patients were produced after the initiation of treatment, and consequently the fungal burden in these samples was mostly low, with a maximum of 374 copies at day two. Helweg-Larsen et al. [14] examined oral washes from HIV patients with and without PCP, and found a lower copy number in asymptomatic patients compared with PCP patients. Larsen et al. [15] were also able to detect PJ in oral washes for patients with PCP, with sensitivity lost if specimens were obtained a few days after treatment was commenced.

Since PJ still cannot be cultured, attempts have been made to predict resistance using genetic markers but with mixed results [36]. Sequential measurements of copy number, as in this study, might be used as a surrogate marker to estimate the adequacy of treatment, and sensitivity to the drug used.

### Strengths and weaknesses

#### Weaknesses

We were only able to include a small number of Danish HIV infected patients with viremia and a low CD4 count. The prevalence of colonization in this group is thus less certain. Secondly, only a few PCP patients were included, with most patients examined on treatment. This will reduce the copy number rapidly, and hampered our ability to define a realistic cut off value with which to distinguish PJ colonization from PCP.

#### Strengths

The quantitative PCR assay developed is specific, has a high sensitivity and large dynamic range. Compared to previous studies, we have systematically and directly compared the prevalence of colonization in disparate groups of immunosuppressed patients. A large number of HIV infected patients from Guinea Bissau at risk for PCP were enrolled, giving a more precise estimate of the prevalence of colonization with PJ.

## Conclusion

In conclusion, we have developed a sensitive quantitative PCR assay with a large dynamic range and applied the method to characterize the level of carriage in different groups of asymptomatic immunosuppressed patients and the dynamics in patients with PCP using oral wash. Carriage was most frequently seen in renal transplanted patients within the first 6 months after operation and in Danish HIV infected patients with viremia combined with low CD4 count, whereas carriage was less frequent among untreated HIV infected patients from Guinea Bissau and patients on immunosuppressive therapy. A fungal load of >1000 copies was suggestive of PCP, whereas a copy number below 30 was seen in asymptomatic patients, who did not develop symptomatic disease on follow up. A grey zone was seen between these 2 extremes suggesting that the method should be used with caution until larger studies have been undertaken.

A higher copy number and a rapid drop of PJ were seen in patients diagnosed with PCP. Thus, the method is potentially useful to evaluate response and drug resistance in PCP patients on treatment.

## Supporting information

S1 Table Correlation between Cp value and DNA copies. (PDF)

S2 Table Interassay variation of the PCR reaction. (PDF)

S1 Fig Relation between Cq and gene copies. (DOCX)
