# Supplementary material for: Detection of Pneumocystis jirovecii in oral wash from immunosuppressed patients as a diagnostic tool
Source: PLoS One. 2017 Mar 30;12(3):e0174012. doi: 10.1371/journal.pone.0174012 (PMC5373571; doi:10.1371/journal.pone.0174012)
Supplement: S1 Table — (PDF) [file pone.0174012.s001.pdf]

copies/PCR reaction	3,00E+00	3,00E+01	3,00E+02	3,00E+03	3,00E+04	3,00E+05	3,00E+06	3,00E+07
replicate 1	11,2	15	18,6	22,8	26,1	29,2	32,4	36,4
replicate 2	11,5	15	18,7	22,9	26,1	29,2	32,7	36,3
replicate 3	11,3	14,7	18,5	22,8	25,7	29,2	32,5	36,7
replicate 4	11,1	14,8	18,5	22,6	25,6	29,1	32,6	37,1
mean	11,3	14,8	18,6	22,8	25,9	29,2	32,5	36,6
st. dev.	0,1	0,1	0,1	0,1	0,2	0	0,1	0,3
Cv	1,3	0,8	0,6	0,5	0,8	0,2	0,4	0,9
Median	11,25	14,9	18,55	22,8	25,9	29,2	32,55	36,55
IQR	0,175	0,225	0,125	0,075	0,425	0,025	0,15	0,425

Table 1: The table shows the correlation of Cp value and DNA copies pr PCR reaction. Mean, standard deviation and Cv is shown in the table. In addition, the median and interquartile ranges and interquartile ranges are shown
