# Supplementary material for: Detection of Pneumocystis jirovecii in oral wash from immunosuppressed patients as a diagnostic tool
Source: PLoS One. 2017 Mar 30;12(3):e0174012. doi: 10.1371/journal.pone.0174012 (PMC5373571; doi:10.1371/journal.pone.0174012)
Supplement: S2 Table — (PDF) [file pone.0174012.s002.pdf]

Interassay variation of the PCR reaction

| | sample 1 | sample 2 | sample 3 | sample 4 | sample 5 | sample 6 | sample 7 | sample 8 |
|---|---|---|---|---|---|---|---|---|
| run 1 | 767 | 3710 | 23 | 848 | 3470 | 31 | 21 | 108000 |
| run 2 | 755 | 3610 | 26 | 833 | 3370 | 34 | 24 | 112000 |
| run 3 | 659 | 3350 | 19 | 730 | 3120 | 25 | 18 | 117000 |
| run 4 | 812 | 3780 | 29 | 894 | 3530 | 37 | 26 | 109000 |
| run 5 | 865 | 3910 | 65 | 949 | 3660 | 76 | 62 | 106000 |
| mean | 772 | 3672 | 32 | 851 | 3430 | 41 | 30 | 110400 |
| std. dev. | 68,3 | 188,3 | 16,6 | 72,7 | 181,2 | 18,2 | 16,1 | 3826 |
| Cv | 8,9 | 5,1 | 51,3 | 8,5 | 5,3 | 44,8 | 53,4 | 3,5 |
| median | 767 | 3710 | 26 | 848 | 3470 | 34 | 24 | 109000 |
| IQR | 57 | 170 | 6 | 61 | 160 | 7 | 5 | 4000 |

**Tabel 2:** Interassay variation of eight different patient samples with varying amounts of Pneumocysts. Mean, standard deviation and Cv are shown in the table. In addition, the median and interquartile ranges are shown
