# Supplementary material for: Detection of Pneumocystis jirovecii in oral wash from immunosuppressed patients as a diagnostic tool
Source: PLoS One. 2017 Mar 30;12(3):e0174012. doi: 10.1371/journal.pone.0174012 (PMC5373571; doi:10.1371/journal.pone.0174012)
Supplement: S1 Fig — (DOCX) [file pone.0174012.s003.docx]

Appendix fig 1 Relation between Cq and gene copies
